# Lattice or Oxygen-Guided Radiotherapy: What If They Converge? Possible Future Directions in the Era of Immunotherapy

**DOI:** 10.3390/cancers13133290

**Published:** 2021-06-30

**Authors:** Gianluca Ferini, Vito Valenti, Antonella Tripoli, Salvatore Ivan Illari, Laura Molino, Silvana Parisi, Alberto Cacciola, Sara Lillo, Dario Giuffrida, Stefano Pergolizzi

**Affiliations:** 1 REM Radioterapia, Viagrande, I-95029 Catania, Italy; vito.valenti@grupposamed.com (V.V.); antonella.tripoli@grupposamed.com (A.T.); 2 Fondazione IOM, Viagrande, I-95029 Catania, Italy; salvatore.illari@fondazioneiom.it; 3 Dipartimento di Scienze Biomediche, Odontoiatriche e delle Immagini Morfologiche e Funzionali Università di Messina, I-98100 Messina, Italy; lauramolino@pec.ordinemedct.it (L.M.); sparisi@unime.it (S.P.); ccclrt91b21f158z@studenti.unime.it (A.C.); slillo@unime.it (S.L.); stpergolizzi@unime.it (S.P.); 4 Medical Oncology Unit, Mediterranean Institute of Oncology, Viagrande, I-95029 Catania, Italy; dario.giuffrida@grupposamed.com

**Keywords:** lattice radiotherapy, GRID radiotherapy, spatially fractionated radiotherapy, Oxygen guided radiotherapy, abscopal effect, bystander effect, bulky tumors, stereotactic radiotherapy, tumor biology

## Abstract

**Simple Summary:**

Oxygen-guided radiotherapy is a new modality for cancer irradiation. Spatially Fractionated Radiation Therapy allows the treatment of hypoxic tumor areas with high radiation doses. Radiotherapy enhances immunotherapy effectiveness. Abscopal and bystander effects are important radiobiological issues. The aim of this paper was to analyze the recent development of a particular kind of radiation therapy that is based on high-dose delivery in small areas within large tumor masses. We performed a narrative review of the radiobiological rationale behind a potential benefit by using these techniques combined with immunotherapy and employing personalized target definition according to hypoxic areas.

**Abstract:**

Palliative radiotherapy has a great role in the treatment of large tumor masses. However, treating a bulky disease could be difficult, especially in critical anatomical areas. In daily clinical practice, short course hypofractionated radiotherapy is delivered in order to control the symptomatic disease. Radiation fields generally encompass the entire tumor mass, which is homogeneously irradiated. Recent technological advances enable delivering a higher radiation dose in small areas within a large mass. This goal, previously achieved thanks to the GRID approach, is now achievable using the newest concept of LATTICE radiotherapy (LT-RT). This kind of treatment allows exploiting various radiation effects, such as bystander and abscopal effects. These events may be enhanced by the concomitant use of immunotherapy, with the latter being ever more successfully delivered in cancer patients. Moreover, a critical issue in the treatment of large masses is the inhomogeneous intratumoral distribution of well-oxygenated and hypo-oxygenated areas. It is well known that hypoxic areas are more resistant to the killing effect of radiation, hence the need to target them with higher aggressive doses. This concept introduces the “oxygen-guided radiation therapy” (OGRT), which means looking for suitable hypoxic markers to implement in PET/CT and Magnetic Resonance Imaging. Future treatment strategies are likely to involve combinations of LT-RT, OGRT, and immunotherapy. In this paper, we review the radiobiological rationale behind a potential benefit of LT-RT and OGRT, and we summarize the results reported in the few clinical trials published so far regarding these issues. Lastly, we suggest what future perspectives may emerge by combining immunotherapy with LT-RT/OGRT.

## 1. Introduction

The treatment of primary or metastatic bulky non-haematologic tumors can be difficult, and radiation therapy is frequently the only available therapeutic option. Systemic therapies are often unable to effectively penetrate within such large lesions due to an inhomogeneous and inadequate neoangiogenesis that could develop asynchronously compared to the tumor cell growth. On the one hand, this phenomenon delays tumor regression necessary for rapid satisfactory symptom relief with chemotherapy only and, on the other hand, could determine well-oxygenated areas alternating with hypoxic areas that are potentially refractory to damage from ionizing radiation. Furthermore, homogeneous irradiation of such large targets could be unsuitable for nearby healthy tissues, which are likely to be significantly affected by a detrimental dose-volume effect. A workaround used to avoid this effect is to aggressively irradiate only very small partial volumes of bulky tumors while limiting the peripheral target dose within tolerance for the neighboring organs at risk. This approach was recently codified by the LATTICE radiotherapy [1], a recently introduced technique that evolves from a historical one, GRID radiotherapy [2], delivered when the low energy (kV) X-rays irradiation was the only available option. GRID and LATTICE radiotherapy are two different expressions of Spatially Fractionated Radiation Therapy (SFRT) [3]. A renewed interest in these techniques exploded since locally targeted radiotherapy was supposed to be able to elicit a tumoricidal response also in unirradiated cancer areas. It is actually known that some radiotherapy effects could be mediated by abscopal and bystander effects and radiation recall phenomenons [4]. In this scenario LATTICE approach could allow a high dose delivery in limited tumor areas, drawing on its potential immunogenicity rather than an ablative role. This assumption could be even more valuable in the era of immunotherapy. Actually, more and more drugs, such as pembrolizumab, nivolumab, and atezolizumab, were effectively and safely tested in combination with the high radiation doses commonly used during stereotactic radiotherapy (SRT) treatments [5]. A continuous matter of debate among radiation oncologists is how to enhance the host immune response against cancer cells in order to investigate the ability of a local treatment such as radiotherapy to trigger a systemic effect. Moreover, we know that tumor volume, due to irregular growth, is not uniformly well-oxygenated, and hypoxic areas are more resistant to the killing effect of radiation. These volumes generally need higher radiation doses in order to eradicate the repopulation ability of cancer cells. Such doses, if equally delivered to the whole large tumor volume, could negatively affect the therapeutic ratio. An option could be a different radiation dose according to the local distribution of hypoxic areas. This introduces the concept of “oxygen guided radiotherapy”, a new investigational approach not yet tested in large clinical trials with promising therapeutic implications.

Here we present a comprehensive narrative review about LATTICE and Oxygen Guided RadioTherapy (OGRT), focusing on their theoretical mechanisms as inferred by analysis of laboratory findings. We also report related clinical experiences and suggest further implementations in the light of the most recent evidence that shows a synergy between radiotherapy and immunotherapy (IT).

## 2. Lattice Radiotherapy: Concept

**Figure 1:**
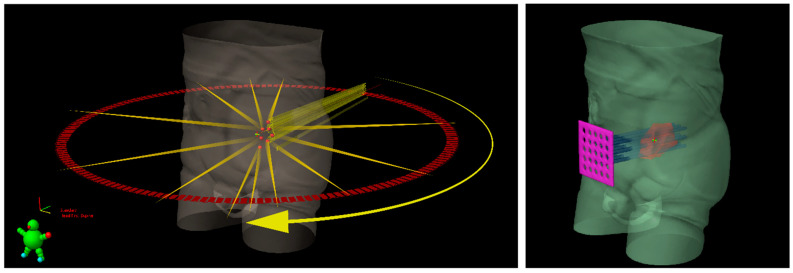
Schematic representation of Lattice radiotherapy with IMRT delivery (on the **left**) and GRID radiotherapy with a sieve-like physical filter (on the **right**) for a bulky tumoral mass, inside which seven high dose spheres (red vertices) are shown.

**Figure 2:**
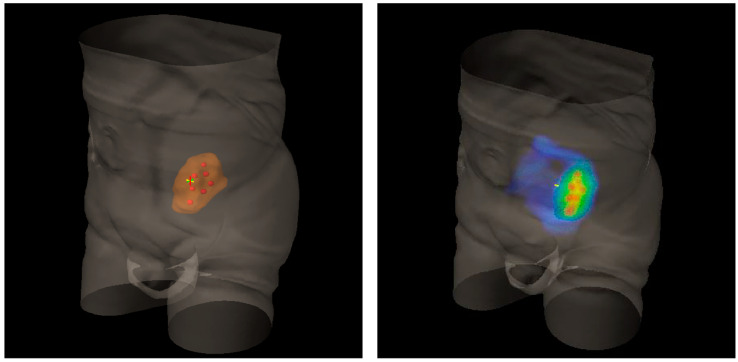
Three-Dimensional (3D) rendering of bulky metastatic iliac lymph node packets from urothelial bladder cancer in a patient candidate for a salvage pelvic radiotherapy (on the **left**). On the **right**, 3D dose distribution with a lattice simulation approach. Blue is for the conventional standard dose, red for high peak ones.

**Figure 3:**
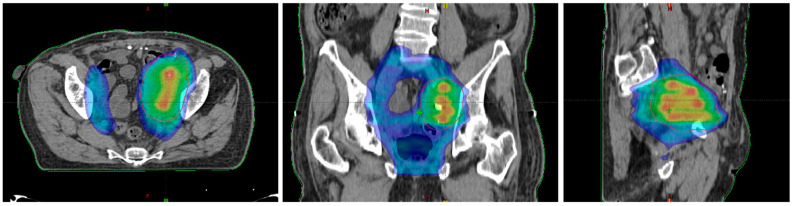
Dose distribution in the axial plane (**left**), the coronal plane (**middle**), and the sagittal plane (**right**).

**Figure 4:**
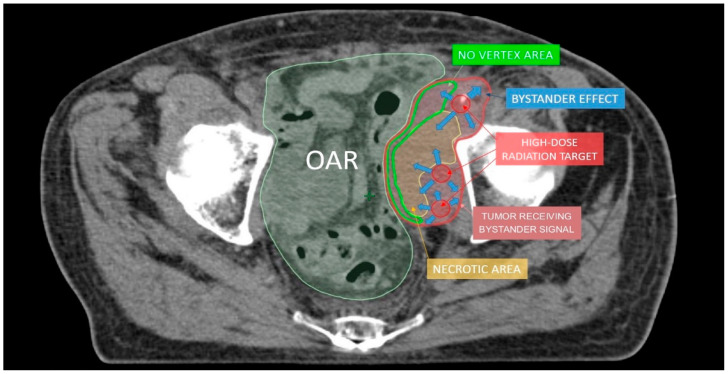
The hypothesis to explain lattice radiotherapy mechanism: pink line delineates global tumor volume, the yellow line shows the necrotic core, and red spheres represent high dose regions and are approximately located at the boundary between the necrotic and normoxic areas (hypoxigenated interface) to radially elicit the bystander effect (blue arrows), accurately avoiding high dose delivery in the green strip due to the hazardous closeness of bowel (Organ At Risk: OAR).

Lattice radiotherapy is the tridimensional evolution of the 2D GRID radiotherapy, a technique used since the beginning of the last century to spare overlying organs at risk, especially the skin while treating large deep-seated tumors at the time of kilovoltage (kV) and 2D-imaging-guided radiotherapy. As a matter of fact, bulky masses could be very difficult to adequately treat due to the fact that the larger the irradiated tumor volume, the larger the radiation dose delivered to neighboring organs at risk (dose-volume effect). This effect could be amplified by the use of kV photons, characterized by low tissue penetrance, so that pursuing an adequate energy deposit at the tumor depth entails a high dangerous dose at the skin surface. GRID approach overcame such a critical issue by fragmenting the radiation beam through a multi-perforated screen placed between the X-ray source and the target in the patient. By alternating blocks and holes, this radiation field array generates a two-dimensional dose distribution, characterized by foci of high radiation dose (peaks) separated by low dose areas (valleys). Such a planning method allowed to significantly reduce the integral peripheral target dose delivered at the boundary with healthy tissues while delivering a high lethal radiation dose to a large portion of tumor volume. The interest in GRID radiotherapy rapidly decreased with the implementation of the newer linear accelerator (LINAC) equipped with much more clinically versatile megavoltage (MV) photons and supported by more and more performing Treatment Planning Systems (TPS) [6]. The historically reported dramatic tumor responses and the chronic difficulties to effectively treat far-advanced bulky tumors recently renewed attention toward the potential curative role of SFRT. Generally, the peak-to-valley dose distribution of physical GRID block radiotherapy is two-dimensional, given the fact that the radiation dose absorption within tumors varies depending on depth, due to a natural photon beam attenuation: this fact could determine an excessive injurious dose deposit in organs at risk beyond the radiotherapy target. This problem can be effectively solved by employing a 3D MultiLeafCollimator-based GRID technique to simulate the equally spaced holes of a physical GRID bloc: such a solution develops a uniform multi-cylinder-shaped dose distribution within a tumor grid pattern directly generated by means of MultiLeafCollimator (MLC) from each gantry angle used, so as to reduce the risk for adjacent organs [3]. The most commonly used diameters of peak dose regions are about 1–1.25 cm wide with spacing (center-to-center distance) of about 1.5–2 cm. Actually, in experimental animal models with a miniaturized lattice method (that is, synchrotron microbeam radiation therapy, MRT, characterized by peaks and valleys at the micron scale), such technique has been proved to be effective in producing a differential biological response between normal and tumor tissues: preserving the first while destroying the second ones [7,8]. Additionally, some authors proved that a high radiation dose delivered in vitro by MRT only in correspondence to the ultra-narrow peak regions is biologically equivalent to a broadly uniform lower dose distribution [9]. The valley-to-peak dose ratio quantifies the dose heterogeneity within the tumor: the lower its value, the lower dose impact on the skin surface and nearby critical structures. MLC-based GRID-like radiation field is more flexible than the 2D-GRID one that needs the creation of a new physical block to be mounted onto the LINAC head for each case. However, the possible inter-leaf radiation leakage could worsen the valley-to-peak dose ratio and the surface dose compared to block-based GRID technique, in view of better control of dose delivery for 3D-MLC GRID radiotherapy [3]. The prescribed dose range is similar to that one used for SRT, generally >15 Gy in a single fraction or a biological equivalent if fractionated, since lower doses could be ineffective to achieve a satisfactory decrease in tumor volume [10]. Lattice radiotherapy could be considered a novel development of GRID radiotherapy, in which geometrically rigid spatial dose fractionation is replaced by the positioning within the tumor of high dose spheres, called vertices, representing a three-dimensional arrangement. Each vertex measures 1 to 2 cm in diameter and is 2–3 cm far from the next ones. Lattice technique is able to produce the same tumor responses and beneficial effects on Organs At Risks (OARs) as the GRID one, but it does not require specific equipment, as its delivery can be done with common LINACs for Intensity Modulated Radiation Therapy (IMRT) (Figure 1) [1]. High-dose Lattice RT could determine a significant tumor volume reduction, even more than expected with a homogeneous dose delivery. This phenomenon seems mediated by the interaction between the killed irradiated tumor cells and the ones nearby unirradiated or those at most impacted by a low radiation dose [11]. The irradiated cancer cell signaling could trigger the tumoricidal effect in unirradiated contiguous tumor subvolumes (bystander effect) and in distantly located metastases (abscopal effect) [12]. These pathways are consequential to the cell release of some interleukins and cytokines or other humoral mediators (e.g., IL-6, IL-8, TGFβ, TNFα, and reactive oxygen and nitrogen species). Microvascular changes could also play a key role in promoting tumor regression via the pro-apoptotic signal derived from the radiation-induced hydrolysis of endothelial cells membrane’s sphingomyelin into ceramide. However, some evidence suggests the fundamental role of lymphocyte recruitment, especially CD8+ cells, in inducing immunomodulated response against cancer cells when lattice radiotherapy is used. Lymphocytes are notoriously radiosensitive, even if slight differences exist among several subpopulations of T cells as compared to B cells [13], and tumor infiltration could be hindered by homogeneous irradiation up to delay the immune response activation mechanisms: this could explain why tumor downsizing is greater with SFRT. On the other hand, from the spared islands of overlying healthy tissues within the radiation field, the migration of surviving cells can start to repair the adjacent damaged areas [3]. Zhang et al. and Wu et al. [1,6,14] efficiently summarize clinical and technical indications and treatment planning parameters for lattice radiotherapy delivery. Such a radiotherapy technique was mostly employed in a palliative setting to debulk large unresectable tumors or for boosting, prior to a normofractionated open-field uniform External Beam RT (EBRT). Dose distribution for a lattice approach (compared with a GRID one) is depicted in Figure 2 and Figure 3. Figure 4 provides a rational explanation of the lattice radiotherapy mechanism. The following paragraph summarizes the results of a clinical application of 3D-Lattice radiotherapy.

## 3. Clinical Use of LATTICE Radiotherapy

The clinical experiences with Lattice radiotherapy are isolated, mostly collected in recent small case series or even individual case reports. The very first was reported in 2010 by Amendola et al. [15]; it was about a large squamous cell carcinoma of the cervix (915 cc) homogeneously irradiated with 1.8 Gy/day fractions up to 36 Gy simultaneously to a hypofractionated boost of 2.4 Gy/fraction/20 fractions (total dose 48 Gy) prescribed to fifteen vertices within the target volume. This treatment resulted in a complete clinical and pathological response, as documented after surgery. Later, the same authors reported a successful tumor regression over 70% of the initial volume of a large ovarian cancer (1495 cc): in this case, the radiation dose delivered to twelve vertices was 27 Gy in three consecutive fractions, followed by a conventionally fractionated dose to the entire tumor volume [16]. The same approach was also effectively used for a locally advanced Pancoast-like non-small cell lung cancer (NSCLC); a single fraction of 18 Gy to three vertices followed by conventional radiotherapy with concomitant chemotherapy [17]. Such a dose prescription was subsequently extended to some further nine bulky NSCLC patients that developed a mean tumor shrinkage equal to 42% [18]. A similar schedule was employed among ten patients with advanced bulky cervical cancer, resulting in a sustained local control as confirmed by morphological and functional post-therapy imaging (MRI and PET) and anticipated by intrafraction Cone Beam CT [19]. Globally, no patient experienced severe lattice RT-related toxicities or death. The feasibility of lattice RT delivery in an SRT-like manner that also incorporates the SBRT dose constraints suggested by AAPM task group 101 [20] was demonstrated for eleven patients with large tumors (up to 4440 ccs) of various histologies and locations [21]. All above lattice treatments were planned with VMAT and commercially available technology equipment. The vertices were positioned deeply away from critical structures so as to ensure a steep dose fall-off from hot peaks to the target periphery. Currently, Lattice radiotherapy is mainly employed and investigated for palliative purposes (NCT04133415), although its usefulness cannot be excluded in other scenarios. Indeed, the attractive capability of concentrating a high radiation dose in small deep tumor subvolumes while keeping a well-tolerated peripheral target dose has promoted the use of such a technique also in radically curative settings. For example, an upfront ablative boost delivered to MRI highly suspicious areas in localized prostate cancer through a lattice technique has proven to be effective and safe in a phase I trial, not adding any further toxicity than expected from a conventional fractionated approach [22]. Another trial by the same authors aims to learn about any difference in terms of cancer-specific outcomes and health-related quality of life between prostate cancer patients treated with upfront lattice boost followed by conventional EBRT or with daily moderately hypofractionated radiotherapy (NCT02307058), attempting to improve the therapeutic ratio of this treatment alternative to surgery [23]. Similarly, other authors explored the use of GammaPod-based Lattice RT for treating large bulky breast tumors and reported a dosimetric feasibility study where, against an extremely high dose concentrated at vertices, a very low mean dose to overlying skin (≈1–2 Gy) was obtained. The translation of this therapeutic strategy into clinical practice could also allow the maximization of heart-sparing, and it could pursue a curative intent in such difficult-to-manage cases more safely [24]. Lastly, known that lattice radiotherapy delivery has a potentially immunogenic role, such a technique was promisingly tested in combination with immunotherapy in a case report by Jiang et al.; they checked if the addition of pembrolizumab is able to enhance the bystander immunomodulatory effect induced by a single high dose fraction of Lattice radiotherapy in a case of pluri-metastatic NSCLC with a bulky cutaneous metastasis. Indeed, they surprisingly reported a complete local response after combined treatment [25]. All the above-mentioned experiences are summarized in Table 1.

## 4. Oxygen-Guided Radiotherapy: Oxygen Is the Needed Comburent for Radiotherapy, Not Only for Fire

The inhomogeneous neoangiogenic sprouting that supports the sprawling cancer cells proliferation could generate hypoxic areas due to a non-uniform and deficient oxygen supply for the entire tumor volume [26]. Such hypoxic “islands” are characterized by slow metabolism and, consequently, by some radioresistance [27]. Within bulky tumors, hypoxic and well-oxygenated clonal strains coexist. To overcome this issue, two approaches are available to radiation oncologists: (1) irradiating the whole tumor tissue with a homogeneously high lethal dose, (2) boosting only the hypoxic subvolumes while not exceeding the threshold dose to kill the well-oxygenated cell clones. The first approach, although effectively cancer-fighting, could be detrimental for nearby healthy tissues in bulky tumors’ treatment due to a deleterious dose-volume effect. The second option requires diversifying the radiation dose within the tumor volume on the basis of its oxygen landscape. This radiation dose delivery method would result in an increase in therapeutic ratio: enhancing the cancer cells death by selective boosting and, simultaneously, reducing radiation-related adverse events thanks to lower radiation exposure of healthy tissues. For this reason, various instrumental tools are being studied to identify ischemic areas within tumors, so introducing the Oxygen Guided Radiation Therapy (OGRT) era [28]. A further complicating matter is the fact that tumor oxygenation rapidly evolves after radiation administration, both spatially and temporally. Such circumstances could make it difficult to determine the optimal therapeutic window where a better radiotherapy sequence can be exploited. Therefore, new in silico models were proposed to explain intratumoral oxygen dynamics prior to and after radiotherapy while waiting for an in vivo validation before clinical use [29]. Briefly, radiation (4 Gy in such a simulation) removes some normoxic cell layers, improving oxygen and nutrients diffusion towards hypoxic ones. At the next radiation administration, the re-oxygenation so obtained allows overcoming the typical radioresistance of previously hypoxic cell clones after their initial transient survival advantage over normoxic ones. Repeated re-oxygenation phenomenons explain why prolonged standard fractionation could be more effective than a single extremely large fraction if this is not able to kill all hypoxic cells [30]. In agreement with these assumptions, Ruggieri et al. suggest, in a mathematical radiobiological model, a simultaneous integrated boost (SIB) selectively directed to hypoxic subvolumes as being more effective rather than a blindly delivered one for multifractionated schedules [31]. The efficacy of radiotherapy could be further enhanced by the concomitant administration of hypoxia-activated prodrugs, even if not still clinically confirmed [32]. Other solutions were proposed to selectively deliver a high radiation dose to the more radio-resistant hypoxic tumor regions, such as radiolabeled antibodies restrictively targeted to cell receptors expressed in the hypoxic tumoral stroma, and proved to be effective to retard tumor growth in mice experiments [33]. Besides, such a tool could exploit a theranostic ability. Regarding intratumoral hypoxic areas detection, Electron Paramagnetic Resonance (EPR) is still not clinically available, but its therapeutic value has been proven for fibrosarcoma in mice and in preliminary human experiences [34,35]. EPR technique was initially used in vivo to quantify the average oxygen variations inside human tumors during radiotherapy schedule [36]. Moreover, EPR images may direct the location of radiation tumor boosts to enhance tumor cure by discriminating between hypoxic and normoxic regions [37]. EPR findings also confirmed that better tumor oxygenation mediated by drug-induced vasodilation improves tumor radiosensitivity. Lastly, its ability to dynamically distinguish responsive regions (well-oxygenated) and unresponsive ones (hypoxic) early during the radiotherapy course could enable a progressive modulation of dose-painting for a better chance of cure [38]. On the other hand, some PET imaging has already proved to be effective in guiding oxygen-based radiotherapy [39,40,41].

## 5. OGRT in Clinical Practice

Oxygen-Guided Radiation Therapy is still a recently introduced concept without a large-scale clinical experience. This is due to the fact that there is a difficulty in tracing hypoxic tumor areas at high spatial resolution by means of currently available methods for routine clinical application. Actually, most of the experiences with the above technical approach are with experimental animal models, more suitable and less cumbersome for preliminary tests. Electron paramagnetic resonance is a preclinical spectroscopic technique that works similarly to nuclear magnetic resonance but is based on the relaxation time of unpaired or photoexcited electrons rather than proton spin. It involves the use of intravenous injection of oxygen-measuring spin probes whose intratumoral spreading is directly correlated with partial pressure of oxygen (pO₂), thus being able to distinguish hypoxic areas from normoxic ones. Such an imaging method has very high accuracy (1 torr pO₂) and spatial resolution (1 mm) that permit modulating dose delivery according to both spatial and temporal variations of intratumoral oxygen landscape. Their feasibility and potential were effectively tested in a mouse fibrosarcoma model by Epel et al. Through the detection and targeting of hypoxic areas (pO_2_ ≤ 10 torr), they proved that it is possible to double tumor control by a selective boosting (13 Gy) without a significant gain for a homogeneous escalated dose (that is including also well-oxygenated subregions) [28]. In a previous similar experiment by the same authors, EPR pO_2_ image-based hypoxic voxels were grouped in radiation boost spheres, resembling vertices of lattice radiotherapy technique [42]. Furthermore, radiation blocks used by these authors to conform the radiation beam shape for selective boosting of hypoxic subvolumes are conceptually similar to those of the predecessor of lattice radiotherapy, the GRID technique [43]. The possibility of manufacturing 3D-printed compensators to modulate radiation beam attenuation allows a SIB delivery within an IMRT approach, as already experienced by Redler et al. [44]. Tumor oxygen background is not static but ever-changing in reaction to fractionated radiotherapy [45]. As demonstrated in a mouse model of glioma, repeated measurement of the oxygen levels by EPR is able to identify local oxygen variations during radiotherapy, leading the way to a theoretical adaptive dose-painting [46]. Such animal results were confirmed in human melanoma [37]. Hypoxia perturbations could compromise tumor control probability also for proton therapy, in spite of its relative independence from oxygen enhancement at lower energies and high linear energy transfer (LET) values, and need radiation dose adjustments [47]. Two recent reviews focus on the usefulness of tumor oxygen mapping [48,49]. The most common method to identify hypoxic tumor subvolumes in clinical practice is PET-based. Indeed, specific hypoxia tracers, such as ^1^⁸F-fluoromisonidazole (FMISO), ^1^⁸F-flortanidazole (^1^⁸F-HX4), and ^1^⁸F-fluoroazomycin arabinoside (^1^⁸F-FAZA), reflect the spatial distribution of intratumoral oxygen and may help to guide hypoxia-based dose escalation [50,51,52,53]. For example, ^1^⁸F-FAZA PET is able to detect hypoxic subvolumes within a ^1^⁸F-FDG PET-homogeneous NSCLC mass [54]. Among patients with locally advanced head and neck squamous cell carcinoma (LAHNSCC), a SIB (up to 84 Gy) restricted to hypoxic regions at FMISO PET may maintain an equal tumor control probability (TCP) compared to a uniform dose-escalated plan while significantly limiting the normal tissue complication probability (NTCP) of OARs, such as parotid glands [55,56,57]. In addition, the ability to perform PET scan early during radiotherapy course permits to detect re-oxygenated areas for which it is possible to safely de-escalate radiation dose due to the new radiosensitive condition, to OARs’ advantage [58]. A trial (NCT02976051) is ongoing to test if a hypoxic cell sensitizer (nimorazole) may enhance radiosensitivity and, consequently, tumor control of FAZA-avid LAHNSCCs [59]. However, some authors questioned the spatial resolution of PET-imaging and attempted to improve it by the use of two combined tracers [60]. MR imaging, particularly functional sequences, could also guide dose painting. Diffusion-weighted images, according to microscopic mobility of water in the cellular environment, are able to indirectly highlight the packing density of tumor cells and to address radiation boost where antiproliferative hypoxia induces a lower cellular density. Similarly, dynamic contrast-enhanced (DCE) MR imaging could distinguish hypovascular tumor subvolumes with an inadequate blood flow for a sufficient oxygen supply [61]. Other MR methods were described for preclinical use by Krishna et al. [62]. Multiparametric MR, including blood oxygen level-dependent hypoxia imaging (BOLD), may identify the amount of hypoxic fraction in recurrent cervical cancer [63]. Finally, some forms of MR imaging are able to monitor radiotherapy-induced changes in tumor hypoxia and, in this way to guide dose modulation [64]. Indeed, BOLD and Tissue Oxygen Level Dependent (TOLD) MR signals correlate with tumor pO₂ and predict tumor growth delay in response to irradiation [65]. Finally, the use of ultrasmall superparamagnetic iron oxide (USPIO) nanoparticles as MR contrast agents [66] combined with quantitative blood-oxygen-level-dependent (qBOLD) [67] imaging for hypoxia and vascular architecture mapping for neovascularization will permit to well evaluate ‘oxygen maps’ within neoplasms. Clinical applications of OGRT are synthesized in Table 2.

## 6. High Dose per Fraction Radiotherapy and Immunotherapy: The Most Recent Evidence for a Successful Cooperation

It is widely acknowledged that high radiation doses can increase the immune response against cancer cells when given with immune checkpoint inhibitors [68,69,70]. Such a dose range is mainly useful for ablative purposes [71,72,73]. In this scenario, irradiated cancer cells are able to execute an escape mechanism from the immune system by expression of molecules, such as PD-L1 or CTLA-4 in NSCL, that need specific blockade by adding exogenous immune regulators, and radiotherapy is able to downregulate PD-L1 expression via the NF-κB [74]. Signatures of antitumoral immune-boosting after high radiation dose delivery (10 Gy per fraction) in oligometastatic breast cancer patients were detected in peripheral blood and expressed as an increase in the amount of CD4+, CD8+ T, and Natural Killer (NK) cells and of pro-inflammatory cytokines, such as IL-1β and TNF-α [75]. Such effects are mediated by the migration of immune cells in tumor tissue since those ones present before RT are extremely radiosensitive and then easily killed [76] or at least inactivated [77]. High radiation doses per fraction in comparison to low ones (6 to 8 Gy vs. 0 to 4 Gy) are able to decrease the immunosuppression elicited by the radiation itself through a reduction of immune-negative regulating granulocytes and monocytes (myeloid-derived suppressor cells, MDSCs) and related signaling (e.g., IL-6, RANTES, and G-CSF) in both on-target and off-target sites, in the latter case enhancing distant antitumor activity, as reported in mice implanted with hepatocellular carcinoma cells [78]. Conversely, similar high doses developed an increase in tumor infiltration by immunosuppressive cells in a mouse model of prostate cancer: this effect was countered by a concomitant rise of functionally active CD8+ T lymphocytes [79]. Following DNA double-strand breaks, high ablative doses seem capable of remapping gene expression of cancer cells, which culminates in the exposure to new antigens, targetable by simultaneously recruited CD8+ and other immune cells. The related T cells priming by tumor-associated dendritic cells could start immune response far from the irradiated disease sites, thus partially explaining the abscopal effect (also known as in situ tumor vaccine), a distant tumor regression triggered by a localized high radiation dose [80]. The fundamental role of this phenomenon was confirmed in a moderately wide and histologically various series of IT+SRT, among which its occurrence was reported up to 29% of cases [81]. In metastatic NSCLC patients, the addition of SRT to immunotherapy also improved the overall response rate (50%) when compared to immunotherapy alone, to an even greater degree considering specific subgroups (PD-L1-negative tumors) [82]. Radio-damaged cancer cells could reduce DNA breaks by specifically activated exonucleases (e.g., TREX1) that can function as a scavenger enzyme, removing these pro-apoptotic signals. It is unclear if coupling DNA repair inhibitors with radio-immunotherapy could increase cancer cell death [83]. The synergistic effect of immunotherapy and stereotactic radiotherapy could modify the prognosis of patients with multiple brain metastasis, especially in some histologies (e.g., melanoma). It is not uncommon to see prolonged survivals without a declining quality of life among these patients. Indeed, they would be treated, as an alternative, with likely memory impairing whole-brain radiotherapy [84]. Such a combination was also investigated in other primitive and metastatic cancers to determine what time sequence (sequential vs. concomitant) and fraction size (hypofractionation vs. conventional fractionation) are better in terms of efficacy and safety, but without conclusive results [85,86,87]. Promising results were also obtained in mice xenografted with glioblastoma multiforme, whereby anti-PD1 antibody plus a 10 Gy single dose offered significantly better results than a single treatment option alone. In this experiment, radiation triggered macrophages repolarization, increasing M1/M2 ratio [88]. Findings concordant with the former ones were reported by Riva et al. that also focused on radiation dosage and fractionation in an attempt to find the more synergistic and effective radio-immunotherapy combinations [89]. The viability of such an approach was also confirmed in human recurrent glioblastoma, especially if enriched by the addition of bevacizumab [90]. The effector function of CD8+ T cells could be boosted by a prodrug formulation of recombinant IL-2 (NKTR-214) that proved to increase the tumor-fighting potential of a 16 Gy single dose, even in contralateral unirradiated tumor site among mice with implanted fibrosarcoma or colorectal cancers [91]. A high radiation dose delivered by means of prostate brachytherapy poses an immune response by increasing TGFβ level and local recruitment of CD4+ and CD8+ T cells, macrophages, and dendritic cells [92]. Such encouraging findings induced to clinically test the feasibility and safety of the combination of prostate brachytherapy with nivolumab; this therapeutic strategy maximized tumor regression in post-radiotherapy core biopsies thanks to a massive immune activation, as evidenced by an increase in immune infiltration and peripheral blood immune cells [93]. Similarly, a large dose per fraction, such as that used in intraoperative radiation therapy (≈20 Gy), gained an aggressive immune response against tumors, overcoming its immune escape mechanisms, among patients with breast cancer [94]. In this case, a continuous balance between antitumor and immunosuppressive mechanisms was also detected, with a clear predominance of the first ones. The combination of anti-PDL1 plus anti-angiogenic therapies with high-dose tumor irradiation could strengthen its antitumor immune effect by subverting the compensatory radio-induced immunosuppressive tumor microenvironment [95]. The highly expressed postradiation TGFβ cytokine is supposed to have an immunosuppressive function that could be targeted by inhibitors to evoke more effective SRT-induced T cell antitumor activity, as evidenced by the CD8+ cells increase and immunosuppressive T regulatory cells decrease in peripheral blood of SRT-treated patients affected by HCC and administered with a well-tolerated TGFβ-specific blockade (galunisertib) [96]. A toll-like receptor 9 agonist was also successfully tested in metastatic lung adenocarcinoma in mice in order to turn off the immunosuppressive effects of high radiation doses (12 Gy × 3 fractions) so as to clearly prevail tumoricidal ones [97]. High radiation doses combined with immunotherapies are able to break through the wall of radioresistance of some tumors, such as renal cell carcinoma, notoriously refractory to conventionally fractionated radiotherapy, and also improve systemic control by producing abscopal effects [98]. Evolving tumor-host interactions in patients treated with high dose radiotherapy and anti-PD1 immunotherapy could be detected by circulating biomarkers, on the basis of which it might be possible to classify patients as responders or non-responders in order to promptly switch the latter to other treatments early [99].

## 7. A Look to the Future and Open Questions

On the basis of this background, the need for dose-painting approaches is evident. Targeting specifically hypoxic areas with an extreme hypofractionation of radiation dose, just as in SRT and LT-RT, could overcome not only the related radioresistance but also trigger intercellular signaling pathways arising as bystander effect [100]. In fact, Prasanna et al. demonstrated that humoral signals extracted from a medium containing 10 Gy-irradiated hypoxic human lung cancer cells added to their unirradiated normoxic counterpart induced a slowdown in the growth of the latter, much more than observed with an analogous well-oxygenated experiment [29]. This finding supports the hypothesis that oxygen could inhibit bystander mechanisms. Thus, we suppose that the vertices positioning of the lattice technique, if guided by hypoxic areas detection, could be more advantageous than a random one. Then again, there is still no consensus for vertices placement in LRT therapy, other than the recommendations to cautiously avoid critical structures and to guarantee a significant drop of the dose at the periphery on the grounds of OARs tolerance [101]. A clinical phase II trial for selectively targeting hypoxic subvolumes in unresectable bulky NSCLC with CT/PET-guided stereotactic high dose irradiation (1–3 fractions each of 10–12 Gy) produced much better results, likely evoked by abscopal and bystander effects than standard chemotherapy and conventional palliative radiotherapy (30 Gy in 10 fractions uniformly delivered to the entire tumor), both in terms of survival outcomes and tumor control, with lower toxicity and improved symptom control [102]. Not only high radiation doses but also low doses could play a key role as antitumoral immunomodulators [103]. Some authors clinically demonstrated that a low dose (1 Gy < doses < 20 Gy in multifractionated regimens) delivery spatially alternating with a high stereotactic one and combined with immunotherapy produces a greater tumor regression than reported in distant no-dose lesions (<1 Gy). Such a finding was shown both for scatter and intentional doses and is supposed to be due to a stromal microenvironment change. In particular, each patient affected by a large tumor burden from different histologies (mostly lung adenocarcinoma) was irradiated with a high dose to a specific site, while off-target peripheral lesions were included in the related scatter area. Alternatively, if low dose coverage was insufficient (<1 Gy), such tumor volumes were intentionally irradiated with a separate isocenter. In order to evaluate the dimensional response rate attributable to low dose effect, at least one no-dose control lesion within the same patient was considered for comparison. Twenty-two of 38 (58%) low-dose (mean 7.3 Gy) lesions compared to 8 of 45 (18%) no-dose lesions developed at least a partial response (4 complete responses vs. 0) with a significant reduction in the longest diameter size (38% vs. 8%). Interestingly, a 5–10 Gy low dose range achieved better results than a 10–15 Gy dose, predicting a window of therapeutic opportunity [104]. Barsoumian et al. hypothesize that high radiation doses prime T cells at the primary tumor location while low ones directed to metastatic sites pave the stroma to react against tumor cells in combination with immunotherapy. Besides macrophage activation and NK cell recruitment, tumor infiltration by CD4+ T cells also plays a pivotal role [105]. The first attempts to integrate tools for modulating hypoxia of tumor microenvironment by the enhanced oxygen-carrying capacity of a specifically assembled hemoglobin with high radiation dose per fraction (8 Gy) and immunotherapy (anti-PD-1 antibody) have already been effectively made in experimental animal models [106]. Then, to optimize radiotherapy effects, the definition of suited time points to exploit tumor oxygen dynamics for adaptive dose-painting in fractionated schedules and to include immunotherapies for reducing the cumulative dose of radiation is required. A biological link between intra-tumoural oxygen landscape and SFRT effects among patients affected by squamous cell carcinomas of the head and neck is investigated by means of hypoxia-specific PET imaging in an ongoing trial (NCT01967927).

On the basis of the above, it is clear the need for defining the more appropriate fraction size of high dose, total one, targeted subvolumes size and shape, time interval between fractions, spatial arrangement of doses, including low ones, radiation beam quality (photons vs. hadrons), and integration with immunotherapy. It is noteworthy to underline that, up to date, there is no clear evidence confirming the advantages of LATTICE radiotherapy over classic irradiation. Indeed, in the absence of comparative analyses, the LATTICE approach should not be considered the standard one. Experimental animal models and preliminary clinical trials are intended to clarify these issues for a standardized therapeutic protocol and to better understand the underlying biological mechanisms.

## Figures and Tables

**Table 1:** Summary of experiences with lattice radiotherapy. Toxicity outcomes reported according to RTOG scale. GI, gastrointestinal; GU, genitourinary.

Authors	Treated Sites (n)	Median Volume (Range) (cc)	Vertices (n)	Patients (n)	Follow-Up Median (Range) (mo)	Histology	Lattice RT Dose/fx (cGy) (Total Dose, Gy)	Further EBRT	Volume Reduction (Range, %)	Side Effects
Amendola et al. [15], 2010	Pelvis	915	15	1	1	Cervix squamous cell carcinoma	240 (48)	Yes (prior)	70%	Diarrhea (G1)
Suarez et al. [16], 2015	Pelvis	1495	12	1	20	Ovarian carcinosarcoma	900 (27)	Yes (post)	70%	None
Amendola et al. [18], 2019	Thorax	175 (46–487)	3	10	6 (1–71)	Non-small cell lung cancer	1800 (18)	Yes (post)	64% (15–83)	Radiation pneumonitis (G1)
Amendola et al. [19], 2020	Pelvis	200.35 (74.1–412.4)	2–11	10	28.5 (4–77)	Squamous cell, adeno/adenosquamous carcinomas	800 (24)	Yes (post)	48% (6–91%)	Diarrhea G1, G2 cystitis
Duriseti S. et al. [21], 2021	Thorax/abdomen/pelvis	687.5 (350–4440)	Ordered threedimensional spatial arrangement	11	-	Various histologies	1334 (66.7)	Yes (simultaneous)	Dosimetric feasibility	
Pollack A et al. [22], 2020	Prostate	-	1–3 (cylinders)	25	66 (21–7)	High-risk prostate cancer	1200–1400 (12–14 Gy)	Yes (post)	-	No acute G3 GU/GI; G1 (15), G2 (4) and G4 (1) (sepsis after a post-treatment transurethral resection) of late GU toxicity; G1 (11) and G2 (4) of late GI toxicity.
Kopchick B. et al. [24], 2020	Breast	-	22–172 (shots)	-	-	-	2000 (20)	-	Dosimetric feasibility	
Jiang L. et al. [25], 2021	Posterior chest wall	63.2	6	1	7	Non-small cell lung cancer	2000 cGy at 69% isodose line	-		None

**Table 2:** Clinical experiences with OGRT. Pt, number of patients; FU, median follow-up (months); H&N, head and neck; OPC, oropharyngeal cancer; NSCLC, non-small cell lung cancer; HNSCC, head and neck squamous cell carcinoma; mpMRI, multi parametric magnetic resonance imaging; MRI, magnetic resonance imaging; SIB, simultaneous integrated boost; TCP, tumor control probability, NTCP, normal tissue complication probability; UTCP, uncomplicated tumor control probability; BOLD, blood oxygen level dependent.

Authors	Tracer	Technique	Site	Histology	Pt	Aims	FU (Median mo)	Results/Toxicity
Williams B. B. et al., 2010 [36]	India ink as an O_2_ reporter	EPR	Different tumor locations	Various histologies	10	Direct measurements of absolute pO_2_ of tumors and other tissues in human subjects	-	-
Stefan Welz et al., 2017 [50]	18F-fluoromisonidazole (FMISO)	dynFMISO PET-CT	H&N	Locally advanced HNSCC	25	Standard radiochemotherapy (stdRT) (70 Gy/35 fractions) vs. DE (77 Gy/35 fractions) with SIB to hypoxic tumor volume (HV)	27	Acute and late toxicity did not show significant differences between the two arms
Lindblom E. et al., 2017 [51]	18F-flortanidazole (18F-HX4)	18F-FMISO-PET	Thorax	Non-small cell lung cancer	10	Delineate hypoxic sub-volumes	-	-
Bollineni, V. R. et al., 2013 [54]	18F-fluoroazomycin arabinoside (18F-FAZA)	PET-CT	Thorax	Advanced-stage non-small cell lung cancer (NSCLC)	11	Detect heterogeneous distributions of hypoxic subvolumes even within homogeneous 18F-FDG background	-	-
Chang, J. H et al., 2013 [55]	18F-fluoromisonidazole (FMISO)	18F-FMISO-PET	H&N	HNSCC	8	PET-guided radiotherapy dose painting to potentially overcome the radioresistant effects of hypoxia in HNSCC	-	Increases the TCP without increasing the NTCP, and increases the UTCP
B. Henriques de Figueiredo et al., 2014 [56]	18F-fluoromisonidazole (FMISO)	18F-FMISO-PET	H&N	III and IV H&N	10	Non-invasive assessment of hypoxia and dose escalation with [18F]-FMISO-PET-guided radiotherapy for head and neck cancers (HNC)	-	Improvement in TCP without excessive increase in NTCP for parotids
Kristi Hendrickson et al., 2011 [57]	18F-fluoromisonidazole (FMISO)	18F-FMISO-PET	H&N	HNSCC	10	PET-guided radiotherapy for boost planning (SIB) to the hypoxic subvolumes	23	Increasing the predicted TCP (mean 17%) without increasing expected complications
Nancy Lee et al., 2016 [58]	18F-FDG and 18F-FMISO	18FDG-PET and dynFMISO PET-CT	OPC	HPV-positive oropharyngeal carcinoma	33	Reducing the dose of radiation based on hypoxia imaging response	32 (21–61)	Intratreatment functional imaging is safe but requires further studies to determine its ultimate role in de-escalation treatment strategies
Abhishek Mahajan et al., 2016 [63]	mpMRI parameters	MRI	Pelvis	Cervix carcinoma	30	Characterizing and detecting vaginal vault/local recurrence	6	Increase the diagnostic accuracy; Hypoxia imaging at follow-up time objectively documents the response; BOLD hypoxia imaging provides information that may be used as a target for radiation dose painting to optimize therapy in the future.

## References

[1] Wu X., Ahmed M.M., Wright J., Gupta S., Pollack A. (2010). On modern technical approaches of three-dimensional high-dose lattice radiotherapy (lrt). Cureus.

[2] Liberson F. (1933). The Value of a Multi-perforated Screen in Deep X-ray Therapy. Radiology.

[3] Pokhrel D., Halfman M., Sanford L., Chen Q., Kudrimoti M. (2020). A novel, yet simple MLC-based 3D-crossfire technique for spatially fractionated GRID therapy treatment of deep-seated bulky tumors. J. Appl. Clin. Med. Phys..

[4] Sindoni A., Severo C., Vadala’ R.E., Ferini G., Mazzei M.M., Vaccaro M., Iatì G., Pontoriero A., Pergolizzi S. (2016). Levetiracetam-induced radiation recall dermatitis in a patient undergoing stereotactic radiotherapy. J. Dermatol..

[5] Schapira E.L., Hubbeling H., Yeap B.Y., Mehan W.A., Shaw A.T., Oh K., Gainor J.F., Shih H.A. (2018). Improved Overall Survival and Locoregional Disease Control with Concurrent PD-1 Pathway Inhibitors and Stereotactic Radiosurgery for Lung Cancer Patients with Brain Metastases. Int. J. Radiat. Oncol..

[6] Wu X., Perez N.C., Zheng Y., Li X., Jiang L., Amendola B.E., Xu B., Mayr N.A., Lu J.J., Hatoum G.F. (2020). The Technical and Clinical Implementation of LATTICE Radiation Therapy (LRT). Radiat. Res..

[7] Crosbie J., Anderson R., Rothkamm K., Restall C.M., Cann L., Ruwanpura S., Meachem S., Yagi N., Svalbe I., Lewis R.A. (2010). Tumor Cell Response to Synchrotron Microbeam Radiation Therapy Differs Markedly from Cells in Normal Tissues. Int. J. Radiat. Oncol..

[8] Bouchet A., Lemasson B., Christen T., Potez M., Rome C., Coquery N., Le Clec’H C., Moisan A., Bräuer-Krisch E., Leduc G. (2013). Synchrotron microbeam radiation therapy induces hypoxia in intracerebral gliosarcoma but not in the normal brain. Radiother. Oncol..

[9] Ibahim M.J., Crosbie J.C., Yang Y., Zaitseva M., Stevenson A.W., Rogers P.A.W., Paiva P. (2014). An Evaluation of Dose Equivalence between Synchrotron Microbeam Radiation Therapy and Conventional Broadbeam Radiation Using Clonogenic and Cell Impedance Assays. PLoS ONE.

[10] Mohiuddin M., Fujita M., Regine W.F., Megooni A.S., Ibbott G.S., Ahmed M.M. (1999). High-dose spatially-fractionated radiation (GRID): A new paradigm in the management of advanced cancers. Int. J. Radiat. Oncol..

[11] Pellizzon A.C.A. (2020). Lattice radiation therapy—its concept and impact in the immunomodulation cancer treatment era. Rev. Assoc. Médica Bras..

[12] Kanagavelu S., Gupta S., Wu X., Philip S., Wattenberg M., Hodge J.W., Couto M.D., Chung K.D., Ahmed M.M. (2014). In VivoEffects of Lattice Radiation Therapy on Local and Distant Lung Cancer: Potential Role of Immunomodulation. Radiat. Res..

[13] Kwan D.K., Norman A. (1977). Radiosensitivity of Human Lymphocytes and Thymocytes. Radiat. Res..

[14] Zhang H., Wu X., Zhang X., Chang S.X., Megooni A., Donnelly E.D., Ahmed M.M., Griffin R.J., Welsh J.S., Ii C.B.S. (2020). Photon GRID Radiation Therapy: A Physics and Dosimetry White Paper from the Radiosurgery Society (RSS) GRID/LATTICE, Microbeam and FLASH Radiotherapy Working Group. Radiat. Res..

[15] E Amendola B., Perez N., Amendola M., Wu X., Ahmed M.M., Iglesias A.J., Estape R., Lambrou N., Bortoletto P. (2010). LATTICE Radiotherapy with RapidArc for Treatment of Gynecological Tumors:Dosimetric and Early Clinical Evaluations. Cureus.

[16] Suarez J.M.B., E Amendola B., Perez N., Amendola M., Wu X. (2015). The Use of Lattice Radiation Therapy (LRT) in the Treatment of Bulky Tumors: A Case Report of a Large Metastatic Mixed Mullerian Ovarian Tumor. Cureus.

[17] Amendola B.E., Perez N., Wu X., Suarez J.M.B., Lu J.J., Amendola M. (2018). Improved outcome of treating locally advanced lung cancer with the use of Lattice Radiotherapy (LRT): A case report. Clin. Transl. Radiat. Oncol..

[18] E Amendola B., Perez N.C., Wu X., Amendola M.A., Qureshi I.Z. (2019). Safety and Efficacy of Lattice Radiotherapy in Voluminous Non-small Cell Lung Cancer. Cureus.

[19] Amendola B.E., Perez N.C., Mayr N.A., Wu X., Amendola M. (2020). Spatially Fractionated Radiation Therapy Using Lattice Radiation in Far-advanced Bulky Cervical Cancer: A Clinical and Molecular Imaging and Outcome Study. Radiat. Res..

[20] Benedict S.H., Yenice K.M., Followill D., Galvin J.M., Hinson W., Kavanagh B., Keall P., Lovelock M., Meeks S., Papiez L. (2010). Stereotactic body radiation therapy: The report of AAPM Task Group 101. Med. Phys..

[21] Duriseti S., Kavanaugh J., Goddu S., Price A., Knutson N., Reynoso F., Michalski J., Mutic S., Robinson C., Spraker M.B. (2021). Spatially fractionated stereotactic body radiation therapy (Lattice) for large tumors. Adv. Radiat. Oncol..

[22] Pollack A., Chinea F.M., Bossart E., Kwon D., Abramowitz M.C., Lynne C., Jorda M., Marples B., Patel V.N., Wu X. (2020). Phase I Trial of MRI-Guided Prostate Cancer Lattice Extreme Ablative Dose (LEAD) Boost Radiation Therapy. Int. J. Radiat. Oncol..

[23] Ferini G., Pergolizzi S. (2021). A Ten-year-long Update on Radiation Proctitis Among Prostate Cancer Patients Treated With Curative External Beam Radiotherapy. In Vivo.

[24] Kopchick B., Xu H., Niu Y., Becker S., Qiu X., Yu C. (2020). Technical Note: Dosimetric feasibility of lattice radiotherapy for breast cancer using GammaPod. Med. Phys..

[25] Jiang L., Li X., Zhang J., Li W., Dong F., Chen C., Lin Q., Zhang C., Zheng F., Yan W. (2021). Combined High-Dose LATTICE Radiation Therapy and Immune Checkpoint Blockade for Advanced Bulky Tumors: The Concept and a Case Report. Front. Oncol..

[26] Castorina P., Castorina L., Ferini G. (2021). Non-Homogeneous Tumor Growth and Its Implications for Radiotherapy: A Phenomenological Approach. J. Pers. Med..

[27] Borkenstein K., Levegrün S., Peschke P. (2004). Modeling and computer simulations of tumor growth and tumor response to radiotherapy. Radiat. Res..

[28] Epel B., Maggio M.C., Barth E.D., Miller R.C., Pelizzari C.A., Krzykawska-Serda M., Sundramoorthy S.V., Aydogan B., Weichselbaum R.R., Tormyshev V.M. (2019). Oxygen-Guided Radiation Therapy. Int. J. Radiat. Oncol..

[29] Prasanna A., Ahmed M.M., Mohiuddin M., Coleman C.N. (2014). Exploiting sensitization windows of opportunity in hyper and hypo-fractionated radiation therapy. J. Thorac. Dis..

[30] Kempf H., Bleicher M., Meyer-Hermann M. (2015). Spatio-Temporal Dynamics of Hypoxia during Radiotherapy. PLoS ONE.

[31] Carlson D.J., Keall P., Loo B.W., Chen Z.J., Brown J.M. (2011). Hypofractionation Results in Reduced Tumor Cell Kill Compared to Conventional Fractionation for Tumors with Regions of Hypoxia. Int. J. Radiat. Oncol..

[32] Hamis S., Kohandel M., Dubois L., Yaromina A., Lambin P., Powathil G.G. (2020). Combining hypoxia-activated prodrugs and radiotherapy in silico: Impact of treatment scheduling and the intra-tumoural oxygen landscape. PLoS Comput. Biol..

[33] Bolli E., D’Huyvetter M., Murgaski A., Berus D., Stange G., Clappaert E.J., Arnouk S., Antunes A.R.P., Krasniqi A., Lahoutte T. (2019). Stromal-targeting radioimmunotherapy mitigates the progression of therapy-resistant tumors. J. Control. Release.

[34] Šentjurc M., Čemažar M., Serša G. (2004). EPR oximetry of tumors in vivo in cancer therapy. Spectrochim. Acta Part A Mol. Biomol. Spectrosc..

[35] Epel B., Redler G., Tormyshev V., Halpern H.J. (2016). Towards Human Oxygen Images with Electron Paramagnetic Resonance Imaging. Single Mol. Single Cell Seq..

[36] Williams B.B., Khan N., Zaki B., Hartford A., Ernstoff M.S., Swartz H.M. (2010). Clinical Electron Paramagnetic Resonance (EPR) Oximetry Using India Ink. Adv. Exp. Med. Biol..

[37] Epel B., Krzykawska-Serda M., Tormyshev V., Maggio M.C., Barth E.D., Pelizzari C.A., Halpern H.J. (2017). Spin Lattice Relaxation EPR pO2 Images May Direct the Location of Radiation Tumor Boosts to Enhance Tumor Cure. Cell Biophys..

[38] Hou H., Abramovic Z., Lariviere J.P., Sentjurc M., Swartz H., Khan N. (2010). Effect of a Topical Vasodilator on Tumor Hypoxia and Tumor Oxygen Guided Radiotherapy using EPR Oximetry. Radiat. Res..

[39] Servagi-Vernat S., Differding S., Sterpin E., Hanin F.-X., LaBar D., Bol A., Lee J.A., Grégoire V. (2015). Hypoxia-guided adaptive radiation dose escalation in head and neck carcinoma: A planning study. Acta Oncol..

[40] Lazzeroni M., Toma-Dasu I., Ureba A., Schiavo F., Wiedenmann N., Bunea H., Thomann B., Baltas D., Mix M., Stoykow C. (2020). Quantification of Tumor Oxygenation Based on FMISO PET: Influence of Location and Oxygen Level of the Well-Oxygenated Reference Region. Adv. Exp. Med. Biol..

[41] Lee N.Y., Mechalakos J.G., Nehmeh S., Lin Z., Squire O.D., Cai S., Chan K., Zanzonico P.B., Greco C., Ling C.C. (2008). Fluorine-18-Labeled Fluoromisonidazole Positron Emission and Computed Tomography-Guided Intensity-Modulated Radiotherapy for Head and Neck Cancer: A Feasibility Study. Int. J. Radiat. Oncol..

[42] Epel B., Redler G., Pelizzari C., Tormyshev V.M., Halpern H.J. (2016). Approaching Oxygen-Guided Intensity-Modulated Radiation Therapy. Single Mol. Single Cell Seq..

[43] Epel B., Maggio M., Pelizzari C., Halpern H.J. (2017). Electron Paramagnetic Resonance pO2 Image Tumor Oxygen-Guided Radiation Therapy Optimization. Chem. Biol. Pteridines Folates.

[44] Redler G., Pearson E., Liu X., Gertsenshteyn I., Epel B., Pelizzari C., Aydogan B., Weichselbaum R., Halpern H.J., Wiersma R.D. (2021). Small Animal IMRT Using 3D-Printed Compensators. Int. J. Radiat. Oncol..

[45] Hou H., Lariviere J.P., Demidenko E., Gladstone D., Swartz H., Khan N. (2009). Repeated tumor pO2 measurements by multi-site EPR oximetry as a prognostic marker for enhanced therapeutic efficacy of fractionated radiotherapy. Radiother. Oncol..

[46] Hou H., Mupparaju S.P., Lariviere J.P., Hodge S., Gui J., Swartz H.M., Khan N. (2013). Assessment of the Changes in 9L and C6 Glioma pO2by EPR Oximetry as a Prognostic Indicator of Differential Response to Radiotherapy. Radiat. Res..

[47] Dahle T.J., Rusten E., Stokkevåg C.H., Silvoniemi A., Mairani A., Fjæra L.F., Rørvik E., Henjum H., Wright P., Boer C.G. (2020). The FLUKA Monte Carlo code coupled with an OER model for biologically weighted dose calculations in proton therapy of hypoxic tumors. Phys. Med..

[48] Bonnitcha P., Grieve S., Figtree G. (2018). Clinical imaging of hypoxia: Current status and future directions. Free. Radic. Biol. Med..

[49] Busk M., Overgaard J., Horsman M.R. (2020). Imaging of Tumor Hypoxia for Radiotherapy: Current Status and Future Directions. Semin. Nucl. Med..

[50] Welz S., Mönnich D., Pfannenberg C., Nikolaou K., Reimold M., la Fougère C., Reischl G., Mauz P.-S., Paulsen F., Alber M. (2017). Prognostic value of dynamic hypoxia PET in head and neck cancer: Results from a planned interim analysis of a randomized phase II hypoxia-image guided dose escalation trial. Radiother. Oncol..

[51] Lindblom E., Dasu A., Uhrdin J., Even A., Van Elmpt W., Lambin P., Wersäll P., Toma-Dasu I. (2017). Defining the hypoxic target volume based on positron emission tomography for image guided radiotherapy—the influence of the choice of the reference region and conversion function. Acta Oncol..

[52] Tran L.-B.-A., Bol A., Labar D., Cao-Pham T.-T., Jordan B., Grégoire V., Gallez B. (2015). Predictive value of 18F-FAZA PET imaging for guiding the association of radiotherapy with nimorazole: A preclinical study. Radiother. Oncol..

[53] Tran L.-B.-A., Bol A., Labar D., Karroum O., Bol V., Jordan B., Grégoire V., Gallez B. (2014). Potential role of hypoxia imaging using 18F-FAZA PET to guide hypoxia-driven interventions (carbogen breathing or dose escalation) in radiation therapy. Radiother. Oncol..

[54] Bollineni V.R., Kerner G.S., Pruim J., Steenbakkers R.J.H.M., Wiegman E.M., Koole M., De Groot E.H., Willemsen A.T., Luurtsema G., Widder J. (2013). PET Imaging of Tumor Hypoxia Using 18F-Fluoroazomycin Arabinoside in Stage III-IV Non-Small Cell Lung Cancer Patients. J. Nucl. Med..

[55] Chang J.H., Wada M., Anderson N.J., Joon D.L., Lee S.T., Gong S.J., Gunawardana D.H., Sachinidis J., O’Keefe G., Gan H.K. (2013). Hypoxia-targeted radiotherapy dose painting for head and neck cancer using18F-FMISO PET: A biological modeling study. Acta Oncol..

[56] De Figueiredo B.H., Zacharatou C., Galland-Girodet S., Benech J., De Clermont-Gallerande H., Lamare F., Hatt M., Digue L., Pujol E.D.M.D., Fernandez P. (2014). Hypoxia imaging with [18F]-FMISO-PET for guided dose escalation with intensity-modulated radiotherapy in head-and-neck cancers. Strahlenther. Und Onkol..

[57] Hendrickson K., Phillips M., Smith W., Peterson L., Krohn K., Rajendran J. (2011). Hypoxia imaging with [F-18] FMISO-PET in head and neck cancer: Potential for guiding intensity modulated radiation therapy in overcoming hypoxia-induced treatment resistance. Radiother. Oncol..

[58] Lee N., Schoder H., Beattie B., Lanning R., Riaz N., McBride S., Katabi N., Li D., Yarusi B., Chan S. (2016). Strategy of Using Intratreatment Hypoxia Imaging to Selectively and Safely Guide Radiation Dose De-escalation Concurrent With Chemotherapy for Locoregionally Advanced Human Papillomavirus–Related Oropharyngeal Carcinoma. Int. J. Radiat. Oncol..

[59] Saksø M., Primdahl H., Johansen J., Nowicka-Matus K., Overgaard J., Dahanca O.B.O. (2019). DAHANCA 33: Functional image-guided dose-escalated radiotherapy to patients with hypoxic squamous cell carcinoma of the head and neck (NCT02976051). Acta Oncol..

[60] Busk M., Horsman M.R., Overgaard J., Jakobsen S. (2019). Dual-tracer PET of viable tumor volume and hypoxia for identification of necrosis-containing radio-resistant Sub-volumes. Acta Oncol..

[61] Van der Heide U.A., Houweling A.C., Groenendaal G., Beets-Tan R.G., Lambin P. (2012). Functional MRI for radiotherapy dose painting. Magn. Reson. Imaging.

[62] Krishna M.C., Matsumoto S., Saito K., Matsuo M., Mitchell J.B., Ardenkjaer-Larsen J.H. (2013). Magnetic resonance imaging of tumor oxygenation and metabolic profile. Acta Oncol..

[63] Mahajan A., Engineer R., Chopra S., Mahanshetty U., Juvekar S., Shrivastava S., Desekar N., Thakur M. (2016). Role of 3T multiparametric-MRI with BOLD hypoxia imaging for diagnosis and post therapy response evaluation of postoperative recurrent cervical cancers. Eur. J. Radiol. Open.

[64] Salem A., Little R.A., Latif A., Featherstone A.K., Babur M., Peset I., Cheung S., Watson Y., Tessyman V., Mistry H. (2019). Oxygen-enhanced MRI Is Feasible, Repeatable, and Detects Radiotherapy-induced Change in Hypoxia in Xenograft Models and in Patients with Non-small Cell Lung Cancer. Clin. Cancer Res..

[65] Hallac R.R., Zhou H., Pidikiti R., Song K., Stojadinovic S., Zhao D., Solberg T., Peschke P., Mason R.P. (2014). Correlations of noninvasive BOLD and TOLD MRI with pO2 and relevance to tumor radiation response. Magn. Reson. Med..

[66] Gkagkanasiou M., Ploussi A., Gazouli M., Efstathopoulos E.P. (2015). USPIO-Enhanced MRI Neuroimaging: A Review. J. Neuroimaging.

[67] Bennani-Baiti B., Pinker K., Zimmermann M., Helbich T.H., Baltzer P.A., Clauser P., Kapetas P., Bago-Horvath Z., Stadlbauer A. (2020). Non-Invasive Assessment of Hypoxia and Neovascularization with MRI for Identification of Aggressive Breast Cancer. Cancers.

[68] He J., Hu Y., Hu M., Li B. (2015). Development of PD-1/PD-L1 Pathway in Tumor Immune Microenvironment and Treatment for Non-Small Cell Lung Cancer. Sci. Rep..

[69] Protopapa M., Kouloulias V., Kougioumtzopoulou A., Liakouli Z., Papadimitriou C., Zygogianni A. (2019). Novel treatment planning approaches to enhance the therapeutic ratio: Targeting the molecular mechanisms of radiation therapy. Clin. Transl. Oncol..

[70] Sindoni A., Minutoli F., Ascenti G., Pergolizzi S. (2017). Combination of immune checkpoint inhibitors and radiotherapy: Review of the literature. Crit. Rev. Oncol..

[71] Owen D., Sio T.T. (2020). Stereotactic body radiotherapy (SBRT) for central and ultracentral node-negative lung tumors. J. Thorac. Dis..

[72] Vadalà R.E., Santacaterina A., Sindoni A., Platania A., Arcudi A., Ferini G., Mazzei M.M., Marletta D., Rifatto C., Risoleti E.V.I. (2016). Stereotactic body radiotherapy in non-operable lung cancer patients. Clin. Transl. Oncol..

[73] Cacciola A., Parisi S., Tamburella C., Lillo S., Ferini G., Molino L., Iatì G., Pontoriero A., Bottari A., Mazziotti S. (2020). Stereotactic body radiation therapy and radiofrequency ablation for the treatment of liver metastases: How and when?. Rep. Pr. Oncol. Radiother..

[74] Chen X., Zhang Q., Luo Y., Gao C., Zhuang X., Xu G., Qaio T. (2016). High-dose irradiation in combination with toll-like receptor 9 agonist CpG oligodeoxynucleotide 7909 downregulates PD-L1 expression via the NF-κB signaling pathway in non-small cell lung cancer cells. OncoTargets Ther..

[75] Muraro E., Furlan C., Avanzo M., Martorelli D., Comaro E., Rizzo A., Fae’ D.A., Berretta M., Militello L., Del Conte A. (2017). Local High-Dose Radiotherapy Induces Systemic Immunomodulating Effects of Potential Therapeutic Relevance in Oligometastatic Breast Cancer. Front. Immunol..

[76] Falcke S.E., Rühle P.F., Deloch L., Fietkau R., Frey B., Gaipl U.S. (2018). Clinically Relevant Radiation Exposure Differentially Impacts Forms of Cell Death in Human Cells of the Innate and Adaptive Immune System. Int. J. Mol. Sci..

[77] Melo A.M., Maher S.G., O’Leary S., Doherty D.G., Lysaght J. (2020). Selective effects of radiotherapy on viability and function of invariant natural killer T cells in vitro. Radiother. Oncol..

[78] Chen J., Wang Z., Ding Y., Huang F., Huang W., Lan R., Chen R., Wu B., Fu L., Yang Y. (2020). Hypofractionated Irradiation Suppressed the Off-Target Mouse Hepatocarcinoma Growth by Inhibiting Myeloid-Derived Suppressor Cell-Mediated Immune Suppression. Front. Oncol..

[79] Lin L., Kane N., Kobayashi N., Kono E.A., Yamashiro J.M., Nickols N.G., Reiter R.E. (2021). High-dose per Fraction Radiotherapy Induces Both Antitumor Immunity and Immunosuppressive Responses in Prostate Tumors. Clin. Cancer Res..

[80] Zhao X., Kang J., Zhao R. (2018). Abscopal effect of radiation on lymph node metastasis in esophageal carcinoma: A case report and literature review. Oncol. Lett..

[81] Trommer M., Yeo S.Y., Persigehl T., Bunck A., Grüll H., Schlaak M., Theurich S., Von Bergwelt-Baildon M., Morgenthaler J., Herter J.M. (2019). Abscopal Effects in Radio-Immunotherapy—Response Analysis of Metastatic Cancer Patients With Progressive Disease Under Anti-PD-1 Immune Checkpoint Inhibition. Front. Pharmacol..

[82] Theelen W.S.M.E., Peulen H.M.U., Lalezari F., Van Der Noort V., De Vries J.F., Aerts J.G.J.V., Dumoulin D.W., Bahce I., Niemeijer A.-L.N., De Langen A.J. (2019). Effect of Pembrolizumab After Stereotactic Body Radiotherapy vs Pembrolizumab Alone on Tumor Response in Patients with Advanced Non–Small Cell Lung Cancer. JAMA Oncol..

[83] Mujoo K., Hunt C.R., Pandita R.K., Ferrari M., Krishnan S., Cooke J.P., Hahn S., Pandita T.K. (2018). Harnessing and Optimizing the Interplay between Immunotherapy and Radiotherapy to Improve Survival Outcomes. Mol. Cancer Res..

[84] Lehrer E.J., McGee H.M., Peterson J.L., Vallow L., Ruiz-Garcia H., Zaorsky N.G., Sharma S., Trifiletti D.M. (2018). Stereotactic Radiosurgery and Immune Checkpoint Inhibitors in the Management of Brain Metastases. Int. J. Mol. Sci..

[85] Sundahl N., Vandekerkhove G., Decaestecker K., Meireson A., De Visschere P., Fonteyne V., De Maeseneer D., Reynders D., Goetghebeur E., Van Dorpe J. (2019). Randomized Phase 1 Trial of Pembrolizumab with Sequential Versus Concomitant Stereotactic Body Radiotherapy in Metastatic Urothelial Carcinoma. Eur. Urol..

[86] Bates J.E., Morris C.G., Milano M.T., Yeung A.R., Hoppe B.S. (2019). Immunotherapy with hypofractionated radiotherapy in metastatic non-small cell lung cancer: An analysis of the National Cancer Database. Radiother. Oncol..

[87] Parisi S., Lillo S., Cacciola A., Santacaterina A., Palazzolo C., Platania A., Settineri N., Franchina T., Tamburella C., Pergolizzi S. (2020). Vaginal Mucosal Melanoma: A Complete Remission after Immunotherapy and ‘0-7-21’ Radiotherapy Regimen (24 Gy/3 fractions/21 days). Folia Med..

[88] Stessin A.M., Clausi M.G., Zhao Z., Lin H., Hou W., Jiang Z., Duong T.Q., Tsirka S.E., Ryu S. (2020). Repolarized macrophages, induced by intermediate stereotactic dose radiotherapy and immune checkpoint blockade, contribute to long-term survival in glioma-bearing mice. J. Neuro-Oncol..

[89] Riva M., Wouters R., Nittner D., Ceuster J., Sterpin E., Giovannoni R., Himmelreich U., Gsell W., Van Ranst M., Coosemans A. (2020). Radiation dose-escalation and dose-fractionation modulate the immune microenvironment, cancer stem cells and vasculature in experimental high-grade gliomas. J. Neurosurg Sci..

[90] Sahebjam S., A Forsyth P., Tran N.D., A Arrington J., Macaulay R., Etame A.B., Walko C.M., Boyle T., Peguero E.N., Jaglal M. (2021). Hypofractionated stereotactic re-irradiation with pembrolizumab and bevacizumab in patients with recurrent high-grade gliomas: Results from a phase I study. Neuro-Oncology.

[91] Walker J.M., Rolig A.S., Charych D.H., Hoch U., Kasiewicz M.J., Rose D.C., McNamara M.J., Hilgart-Martiszus I.F., Redmond W.L. (2019). NKTR-214 immunotherapy synergizes with radiotherapy to stimulate systemic CD8+T cell responses capable of curing multi-focal cancer. J. Immunother. Cancer.

[92] Keam S.P., Halse H., Nguyen T., Wang M., Losio N.V.K., Mitchell C., Caramia F., Byrne D.J., Haupt S., Ryland G. (2020). High dose-rate brachytherapy of localized prostate cancer converts tumors from cold to hot. J. Immunother. Cancer.

[93] Yuan Z., Fernandez D., Dhillon J., Abraham-Miranda J., Awasthi S., Kim Y., Zhang J., Jain R., Serna A., Pow-Sang J.M. (2020). Proof-of-principle Phase I results of combining nivolumab with brachytherapy and external beam radiation therapy for Grade Group 5 prostate cancer: Safety, feasibility, and exploratory analysis. Prostate Cancer Prostatic Dis..

[94] Linares-Galiana I., Berenguer-Frances M.A., Cañas-Cortés R., Pujol-Canadell M., Comas-Antón S., Martínez E., Laplana M., Pérez-Montero H., Pla-Farnós M.J., Navarro-Martin A. (2021). Changes in peripheral immune cells after intraoperative radiation therapy in low-risk breast cancer. J. Radiat. Res..

[95] Chen J.L.-Y., Pan C.-K., Huang Y.-S., Tsai C.-Y., Wang C.-W., Lin Y.-L., Kuo S.-H., Shieh M.-J. (2021). Evaluation of antitumor immunity by a combination treatment of high-dose irradiation, anti-PDL1, and anti-angiogenic therapy in murine lung tumors. Cancer Immunol. Immunother..

[96] Reiss K.A., Wattenberg M.M., Damjanov N., Dunphy E.P., Jacobs-Small M., Lubas M.J., Robinson J., DiCicco L., Garcia-Marcano L., Giannone M.A. (2021). A Pilot Study of Galunisertib plus Stereotactic Body Radiotherapy in Patients with Advanced Hepatocellular Carcinoma. Mol. Cancer Ther..

[97] Younes A.I., Barsoumian H.B., Sezen D., Verma V., Patel R., Wasley M., Hu Y., Dunn J.D., He K., Chen D. (2021). Addition of TLR9 agonist immunotherapy to radiation improves systemic antitumor activity. Transl. Oncol..

[98] Wei Q., He H., Lv L., Xu X., Sun W. (2020). The promising role of radiotherapy in the treatment of advanced or metastatic renal cell carcinoma: A narrative review. Transl. Androl. Urol..

[99] Milhem C., Moralès O., Ingelaere C., Pasquier D., Mordon S., Mortier L., Mirabel X., Delhem N. (2020). Combination of High Dose Hypofractionated Radiotherapy with Anti-PD1 Single Dose Immunotherapy Leads to a Th1 Immune Activation Resulting in a Complete Clinical Response in a Melanoma Patient. Int. J. Mol. Sci..

[100] Asur R., Butterworth K.T., Penagaricano J.A., Prise K.M., Grifn R.J. (2015). High dose bystander efects in spatially fractionated radia-tion therapy. Cancer Lett..

[101] Yan W., Khan M.K., Wu X., Simone C.B., Fan J., Gressen E., Zhang X., Limoli C.L., Bahig H., Tubin S. (2020). Spatially fractionated radiation therapy: History, present and the future. Clin. Transl. Radiat. Oncol..

[102] Tubin S., Khan M.K., Salerno G., Mourad W.F., Yan W., Jeremic B., Tubin S. (2019). Mono-institutional phase 2 study of innovative Stereotactic Body RadioTherapy targeting PArtial Tumor HYpoxic (SBRT-PATHY) clonogenic cells in unresectable bulky non-small cell lung cancer: Profound non-targeted effects by sparing peri-tumoral immune microenvironment. Radiat. Oncol..

[103] De Olza M.O., Bourhis J., Irving M., Coukos G., Herrera F.G. (2020). High versus low dose irradiation for tumor immune reprogramming. Curr. Opin. Biotechnol..

[104] Menon H., Chen D., Ramapriyan R., Verma V., Barsoumian H.B., Cushman T.R., Younes A., Cortez M.A., Erasmus J.J., De Groot P. (2019). Influence of low-dose radiation on abscopal responses in patients receiving high-dose radiation and immunotherapy. J. Immunother. Cancer.

[105] Barsoumian H.B., Ramapriyan R., I Younes A., Caetano M.S., Menon H., I Comeaux N., Cushman T.R., E Schoenhals J., Cadena A.P., Reilly T.P. (2020). Low-dose radiation treatment enhances systemic antitumor immune responses by overcoming the inhibitory stroma. J. Immunother. Cancer.

[106] Sang W., Xie L., Wang G., Li J., Zhang Z., Li B., Guo S., Deng C.X., Dai Y. (2020). Oxygen-Enriched Metal-Phenolic X-Ray Nanopro-cessor for Cancer Radio-Radiodynamic Therapy in Combination with Checkpoint Blockade Immunotherapy. Adv. Sci. (Weinh).

